# Mutational Analysis of *EYA1*, *SIX1* and *SIX5* Genes and Strategies for Management of Hearing Loss in Patients with BOR/BO Syndrome

**DOI:** 10.1371/journal.pone.0067236

**Published:** 2013-06-28

**Authors:** Mee Hyun Song, Tae-Jun Kwon, Hui Ram Kim, Ju Hyun Jeon, Jeong-In Baek, Won-Sang Lee, Un-Kyung Kim, Jae Young Choi

**Affiliations:** 1 Department of Otorhinolaryngology, Kwandong University College of Medicine, Myongji Hospital, Goyang, South Korea; 2 Department of Biology, College of Natural Sciences, Kyungpook National University, Daegu, South Korea; 3 Department of Otorhinolaryngology, Inje University College of Medicine, Goyang, South Korea; 4 Department of Otorhinolaryngology, Yonsei University College of Medicine, Seoul, South Korea; Emory Univ. School of Medicine, United States of America

## Abstract

**Background:**

Branchio-oto-renal (BOR) or branchio-otic (BO) syndrome is one of the most common forms of autosomal dominant syndromic hearing loss. Mutations in *EYA1*, *SIX1* and *SIX5* genes have been associated with BOR syndrome. In this study, clinical and genetic analyses were performed in patients with BOR/BO syndrome focusing on auditory manifestations and rehabilitation.

**Methods:**

The audiologic manifestations were reviewed in 10 patients with BOR/BO syndrome. The operative findings and hearing outcome were analyzed in patients who underwent middle ear surgeries. The modality and outcome of auditory rehabilitation were evaluated. Genetic analysis was performed for *EYA1*, *SIX1*, and *SIX5* genes.

**Results:**

All patients presented with mixed hearing loss. Five patients underwent middle ear surgeries without successful hearing gain. Cochlear implantation performed in two patients resulted in significant hearing improvement. Genetic analysis revealed four novel *EYA1* mutations and a large deletion encompassing the *EYA1* gene.

**Conclusions:**

Auditory rehabilitation in BOR/BO syndrome should be individually tailored keeping in mind the high failure rate after middle ear surgeries. Successful outcome can be expected with cochlear implantations in patients with BOR/BO syndrome who cannot benefit from hearing aids. The novel *EYA1* mutations may add to the genotypic and phenotypic spectrum of BOR syndrome in the East Asian population.

## Introduction

Branchio-oto-renal (BOR) syndrome (OMIM 113650) or branchio-otic (BO) syndrome (OMIM 602588) is one of the most common forms of autosomal dominant syndromic hearing loss with an incidence of 1∶40,000 and is responsible for causing 2% of profoundly deaf children [1]. The clinical manifestations of BOR syndrome include hearing loss (93%), preauricular pits or tags (82%), renal anomalies (67%), branchial fistulae (49%), and pinnae deformity (36%) [2]. Diagnostic criteria proposed by Chang *et al*. [3] in 2004 are most widely used for the clinical diagnosis of BOR syndrome. In 1997, Abdelhak *et al.* [4] reported the human homolog of the *Drosophila eyes absent* gene (*EYA1*) as the causative gene of BOR syndrome and identified novel mutations of the *EYA1* gene in seven families demonstrating typical features of BOR syndrome. Mutations of the *EYA1* gene are found in approximately 40% of patients with BOR syndrome [3]. In addition to the *EYA1* gene, mutations in the *SIX1* and *SIX5* genes have been reported to cause BOR phenotypes, although the pathogenic role of the *SIX5* gene has been questioned recently [5], [6], [7]. *SIX1* mutations have been shown to disrupt the *EYA1*-*SIX1*-DNA complexes [5], [8].

The *EYA1* gene located on chromosome 8q13.3 encodes a transcriptional co-activator required for eye morphogenesis which consists of three isoforms (a, b, c) and four transcript variants (EYA1A–1D) as a result of alternative splicing [9], [10]. EYA1C (transcript variant 3; NM_000503), one of the two variants of isoform b, is the longest transcript encoded by 16 coding exons extending over 156 kb [10].

To date, approximately 160 mutations of the *EYA1* gene have been associated with BOR/BO syndrome, and frameshift or nonsense mutations are the most commonly detected mutations, followed by splice-site and missense mutations [7], [11], [12], [13], [14], [15], [16], [17]. Approximately 20% of the patients with BOR/BO syndrome have been reported to be caused by complex genomic rearrangements of the *EYA1* gene that are not detected by direct sequencing of the coding region [3]. No specific hot spot has been demonstrated for *EYA1* mutations causing BOR syndrome and most of the mutations are unique to individual families [3].

Hearing impairment, the most common phenotypic feature of BOR syndrome, is found in various forms among which mixed type of hearing loss is most frequently reported (50%), followed by conductive (30%) and sensorineural type (20%) [2]. Analysis of computed tomography (CT) imaging of the temporal bone has revealed malformations of the middle and inner ear structures in majority of the patients with BOR syndrome [2], [18]. Chen *et al.* [2] found cochlear hypoplasia (63%), enlarged vestibular aqueduct (46%), bulbous internal auditory canal (25%), and ossicular malposition (50%) or malformation (33%), while Propst *et al.* [18] described hypoplastic apical turn of cochlea (100%), medial deviation of the facial nerve (90.5%), and funnel shaped internal auditory canals (85.7%) as the most common findings of the temporal bone in patients clinically diagnosed as BOR syndrome.

Because of the conductive component of hearing loss identified in most cases of patients with BOR syndrome, attempts have been made to improve hearing through middle ear exploratory tympanotomy and ossicular reconstruction. However, there are only several studies dealing with hearing outcome of exploratory tympanotomy in patients with BOR syndrome mostly reported before the identification of genetic causes of BOR syndrome, which demonstrated unsatisfactory results [19]. Since variable clinical manifestations concerning onset, degree, type, and progressiveness of hearing loss can be seen in BOR syndrome, auditory rehabilitation in these patients have to be carefully evaluated and managed according to individual conditions. In this study, clinical analysis was performed in 10 patients with BOR/BO syndrome focusing on auditory manifestations and rehabilitation, and the results of mutational analysis for the *EYA1*, *SIX1*, and *SIX5* genes are reported.

## Materials and Methods

### Subjects

Seven families (10 patients) including one multiplex family showing hearing loss and one or more of the typical features of BOR syndrome were included in this study for clinical and genetic analyses. The clinical diagnosis as typical BOR syndrome was made when the clinical criteria proposed by Chang *et al.* [3] were satisfied, whereas atypical BOR syndrome was diagnosed when only one or two features of BOR syndrome were present together with hearing loss. The combined anomalies were thoroughly reviewed in all patients; however, the results of renal ultrasonography were available in 4 of 10 patients. Written informed consent was obtained from the participating individuals, and this study was approved by the Institutional Review Board of the Yonsei University College of Medicine.

### Audiologic Evaluation

Serial pure tone audiometries were performed in all patients and the various clinical manifestations regarding hearing loss were carefully reviewed. The type, degree, onset, progressiveness and/or fluctuation of hearing loss were evaluated in each patient. The threshold of pure tone audiometry was defined as the average of thresholds at 500, 1000, 2000, and 3000 Hz. The follow-up period of audiologic evaluations ranged from 3 months to 7.5 years. Speech audiometry and language evaluations were carried out in some of the patients whenever possible.

### Radiologic Evaluation

The temporal bone CT scan was performed in all of 10 patients and temporal magnetic resonance imaging (MRI) was available for analysis in 4 patients. The temporal bone CT scan was performed with a 16 multidetector row CT scanner (Somatom Sensation 16; Siemens, Erlangen, Germany) using a standard temporal bone protocol. Contiguous 0.7-mm scans of the temporal bone were acquired in the axial plane and reformatted coronally with 1.0-mm increments. CT images were performed, digitally stored, and displayed by using the Picture Archiving Communication System (PACS) (Centricity; GE Healthcare, Milwaukee, WI).

MRI was acquired by using a 3.0-T (Achieva; Philips Medical Systems, Best, the Netherlands) or 1.5-T system (Intera; Philips Medical Systems, Best, the Netherlands) with a six-channel sensitivity encoding (SENSE) head coil. The targeted parasagittal scan perpendicular to the long axis of the internal auditory canal was obtained with T2-weighted three-dimensional (3-D) turbo spin-echo (TSE) sequence with driven equilibrium RF reset pulse (DRIVE), following routine MR sequences with spin-echo T1- and T2-weighted images. The sequence parameters for the T2-weighted 3-D FSE sequence with DRIVE were as follows: repetition time (TR)/echo time (TE) = 1500/200 ms, 256 acquisition/256 reconstruction, 15-cm field of view, 1.5-mm section thickness with a 0.75-mm overlap, number of acquisitions = 2, and the scan time was less than 5 minutes.

The morphologies of the cochlea, vestibule, semicircular canals, internal auditory canals, vestibular aqueduct, and middle ear structures were analyzed on temporal bone CT scans. On temporal MRI, abnormalities of the brain, the cochleovestibular and facial nerves, as well as the endolymphatic duct and sac were evaluated.

### Gene Screening

All exons and exons-intron boundaries of *EYA1, SIX1* and *SIX5* gene were amplified by Polymerase Chain Reaction (PCR) with specific primers (Table S1) designed using Primer 3 software (http://frodo.wi.mit.edu/). PCR with H taq polymerase (Solgent, Daejeon, South Korea) proceeded as following cycles: 15 minutes at 95°C, repeated of 30–40 cycles of denaturation at 94°C for 20 seconds; annealing at * °C for 40 seconds; extension at 72°C for 30 seconds (* is depended by melting temperature of primers). Last extension step was performed at 72°C for 5 minutes. Particularly, exons 1 and 2 of *SIX5* were amplified using LA taq (TaKara, Otsu, Shiga, Japan), because these regions have high GC contents. PCR products were separated on 1.5% agarose gel. The PCR products were purified using Shrimp alkaline phosphatase (USB, Cleveland, OH, USA) and exonuclease I at 37°C for 70 minutes and directly sequenced using the Bigdye Terminator v3.1 Cycle Sequencing Kit (Applied Biosystems, Foster City, CA, USA). Ethanol precipitation was used for purification of sequencing reaction products before running the samples on the 3130xl Genetic Analyzer (Applied Biosystems, Foster City, CA, USA). The data was analyzed utilizing Sequencing analysis v5.2 (Applied Biosystems, Foster City, CA, USA) and Chromas Pro v1.5 software (Technelysium, Pty Ltd., Tewantin, QLD, Australia). Multiple alignments of the analyzed sequences were performed using CLC sequence Viewer v.6.0 software (CLC Bio, Aarhus, Denmark).

### Splicing Assays

To analyze the splicing pattern, minigene vector was manufactured to include each exon along with about 300 bp of 5′- and 300 bp of 3′- intronic flanking regions (c.699+5 G>A, c.1140+1 G>A, c.1598-2 G>A). Amplified wild or mutant type products digested with *Bam*HI and *EcoRI* were inserted into multiple cloning sites between exon A and exon B in pSPL3 or pSPL3b vectors. For *in vitro* splicing assay, HeLa cells were cultured in DMEM (containing 10% FBS, 1% Penicillin/Streptomycin) at 37°C in 5% CO_2_ concentration. Prior to transfection, HeLa cells were seeded at a density of 2.7×10^6^ on a 60 mm culture dish. Hybrid minigenes in pSPL3 or pSPL3b vector were transiently transfected into HeLa cells using Fugene 6 Transfection reagent (Promega, Madison, WI, USA) at 3 µL per µg of DNA, and the transfected cells were harvested 24 hours after transfection. Total RNA was extracted from the transfected HeLa cells using an RNeasy Mini Kit (Qiagen, Hilden, Germany) according to the manufacturer’s protocol. Approximately 1 µg of total RNA was reverse-transcribed into cDNA using a High Capacity cDNA Reverse Transcription Kit (Applied Biosystems, Foster City, CA, USA). The cDNA was used as a template for PCR amplification of pSPL3 or pSPL3b vector-specific primers SD6 and SA2. The size of amplified normal and mutant fragments was confirmed on 1.5% agarose gel by electrophoresis.

### Multiplex Ligation-dependent Probe Amplification

Multiplex Ligation-dependent Probe Amplification (MLPA) was performed to detect copy number variations such as deletions or duplications. The SALSA MLPA probemix P153-A2 *EYA1* kit (MRC-Holland, Amsterdam, The Netherlands) that was used includes 17 probes for 14 of the 18 *EYA1* exons (probes for exons 1, 8, 13, 16 are not included whereas two different probes exist for exons 6, 9, 10) and 14 control probes. MLPA was performed according to the manufacturer’s instructions: denaturation at 98°C for 5 minutes; stabilization at 25°C; hybridization at 95°C for 1 minute and at 60°C for 16–20 hours, stabilization at 54°C; ligation at 54°C for 15 minutes, inactivation at 98°C for 5 minutes, and stabilization at 20°C. PCR was carried out as follows: 35 cycles of denaturation at 95°C for 30 seconds; annealing at 60°C for 30 seconds; extension at 72°C for 1 minute. Final extension step was performed at 72°C for 20 minutes. Amplified products containing GeneScan™-500 LIZ® Size Standard (Applied Biosystems, Foster City, CA, USA) and Hi-Di Formamide (Applied Biosystems, Forster City, CA, USA) were separated and quantified by capillary electrophoresis on the 3130xl Genetic Analyzer (Applied Biosystems, Forster City, CA, USA), and the data were analyzed using GeneMarker software v1.6 (Softgenetics, State College, PA, USA).

### Genotype Analysis of Microsatellite Markers

Four microsatellite markers, D8S1795, D8S1060, D8S1807 and D8S570, in the region of 2 Mb including the *EYA1* gene were selected from the NCBI database (www.ncbi.nlm.gov) considering their heterozygosity. PCR with H taq polymerase was performed using fluorescently tagged primers. Two PCR products of different fragment size and 0.1 µL of GeneScan™-500 LIZ® Size Standard (Applied Biosystems, Foster City, CA, USA) were mixed and diluted with 8.9 µL of Hi-Di Formamide (Applied Biosystems, Forster City, CA, USA). Final diluted products were separated and detected by using ABI 3130x genetic analyzer. GeneMapper v4.0 software (Applied Biosystems, Forster City, CA, USA) was used to analyze genotypes for each marker.

## Results

### Clinical Presentations

Seven families including 10 patients were analyzed. All of the patients were females and their age at the time of diagnosis ranged from 1 to 43 years (Table 1). The clinical features identified in each patient are shown in Table 2. Nine patients were diagnosed as typical BOR/BO syndrome according to the criteria by Chang *et al.* [3], while one patient exhibited only mixed type of hearing loss and inner ear anomalies to be classified as atypical BOR/BO syndrome. Of the major criteria other than hearing loss, preauricular pit was the most common finding seen in 80%, followed by branchial anomalies present in 30% of the patients. Patient 6 aged 14 years and patient 10 aged 43 years had received excision of bilateral branchial fistulae at other hospitals in childhood, and demonstrated postoperative scars bilaterally along the anterior border of the sternocleidomastoid muscle. Patient 9 aged 1 year exhibited nondischarging pits also at the anterior border of the sternocleidomastoid muscle bilaterally without any palpable cystic portion. Of the minor criteria, inner ear and middle ear anomalies detected on temporal bone CT were identified in all of the patients, whereas external ear anomalies were observed in only 2 patients.

**Table 1:** Auditory manifestations and management of hearing loss in patients with BOR/BO syndrome.

Families	Patients	Sex/Age*(Yr)	Auditory manifestations				Middle ear surgery	Auditory rehabilitation	
			Type	PTA thresholds (dB HL)	Onset	Progression		HA	CI
I	1	F/2	Mixed	80 (R), 60 (L)	Congenital	No	–	(B)	
	2	F/14	Mixed	65 (R), 90 (L)	Postlingual	No	–	(B)	
	3	F/16	Mixed	60 (R), 75 (L)	Postlingual	No	–	(B)	
	4	F/38	Mixed	90 (R), 85 (L)	Postlingual	No	–	(B)	
II	5	F/12	Mixed	55 (R), 45 (L)	Postlingual	Yes	Ossiculoplasty (R), stapedotomy (R)	(B)	
III	6	F/14	Mixed	65 (R), 65 (L)	Postlingual	Yes	Ossiculoplasty (R)	(B)	CI (R)
IV	7	F/13	Mixed	90 (R), 105 (L)	Postlingual	Yes	–	(B)	CI (R)
V	8	F/9	Mixed	50 (R), 50 (L)	Congenital	No	Ossiculoplasty (L)	(B)	
VI	9	F/1	Mixed	50 (R), 75 (L)	Prelingual	Yes	Cholesteatoma removal (R)	(B)	
VII	10	F/43	Mixed	65 (R), 60 (L)	Postlingual	Yes	Stapedotomy (B)	(B)	

* Age at first visit to our clinic; Yr: year; PTA: pure tone audiometry; dB HL: decibel hearing level; HA = hearing aid; CI = cochlear implantation; R: right, L: left, B: both.

**Table 2:** Clinical features and diagnostic criteria in patients with BOR/BO syndrome.

Clinical Features	Patients									
	1	2	3	4	5	6	7	8	9	10
**Major Critieria**										
Branchial anomalies	–	–	–	–	–	O	–	–	O	O
Deafness	O	O	O	O	O	O	O	O	O	O
Preauricular pits	O	O	O	O	O	O	–	O	O	-
Renal anomalies*	?	?	?	?	?	–	?	–	O	–
**Minor Criteria**										
External ear anomalies	O	–	–	–	O	–	–	–	–	–
Middle ear anomalies	O	O	O	O	O	O	O	O	O	O
Inner ear anomalies	O	O	O	O	O	O	O	O	O	O
Preauricular tags	–	–	–	–	–	–	–	–	–	–
Other: facial asymmetry etc.	–	–	–	–	–	–	–	–	–	–
**Diagnosis**										
3 Major						O			O	
2 Major +2 Minor	O	O	O	O	O			O		O
Typical/Atypical	Typical	Typical	Typical	Typical	Typical	Typical	Atypical	Typical	Typical	Typical

* Evaluated for renal anomalies only in patients 6, 8, and 10;

?: renal ultrasonography not performed.

Results of renal manifestations were evaluated in 4 of 10 patients. In one patient (patient 9), a tiny cyst was seen in the right renal cortex and mild pelvic dilatation of the right kidney was identified by renal ultrasonography without any evidence of renal dysfunction. The other three patients did not reveal any abnormality on renal ultrasonography or blood testing.

### Radiologic Findings and Temporal Bone Anomalies

**Figure 1:**
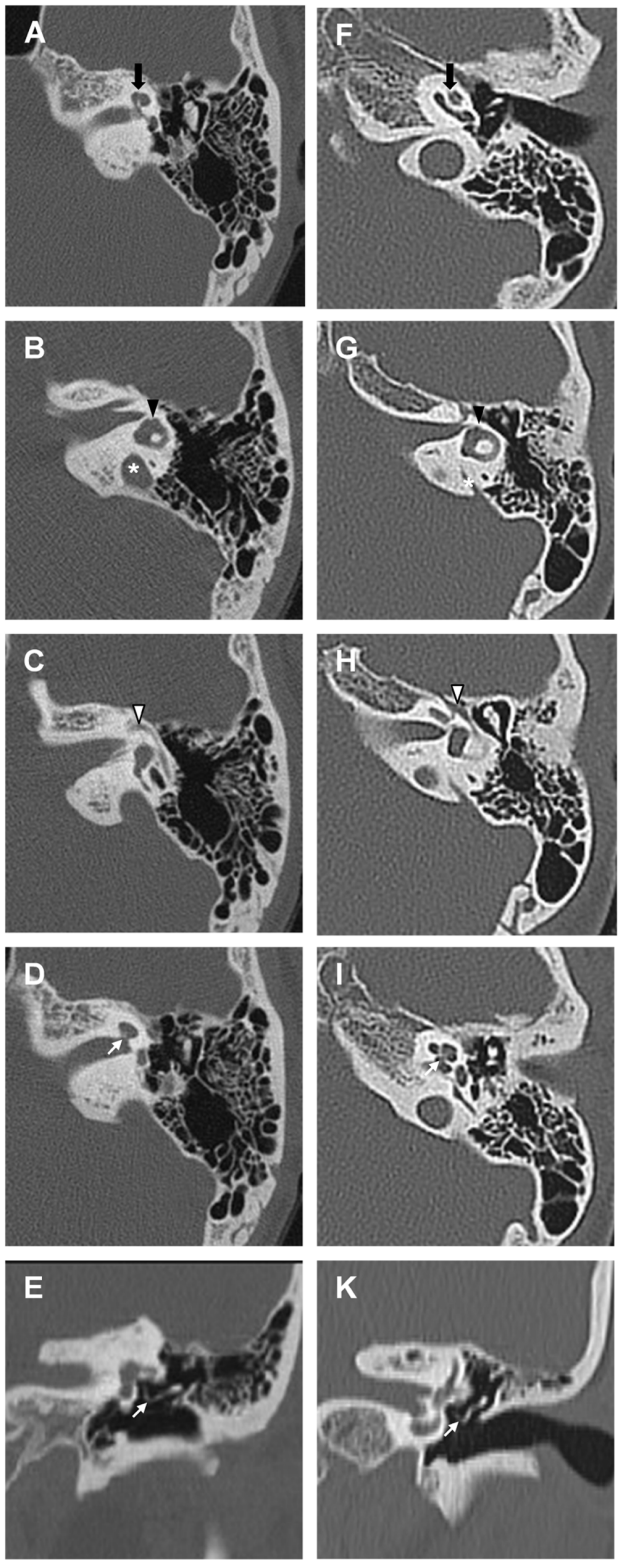
The findings of temporal bone CT in patient 7 and normal control. (A–E) These images are temporal bone CT in patient 7. (A) Cochlear hypoplasia type III with less than two turns is indicated by a black arrow. (B) The vestibular aqueduct (white asterisk) was enlarged and seen in a circular shape in the axial view. The lateral semicircular canal was slightly hypoplastic and the vestibular was dilated (black arrowhead). (C) The facial nerve ran inferior to the hypoplastic cochlea and displayed an obtuse angle between the labyrinthine and tympanic segments (white arrowhead). (D) The modiolus (white arrow) was present but defective and hypoplastic. (E) The ossicular chain (white arrow) seen in the coronal view was positioned in a different angle compared to the normal control. (F–K) These images are temporal bone CT in normal control.

All of the patients with BOR/BO syndrome underwent high resolution temporal bone CT, and temporal MRI was available for analysis in four of the patients (Table 3). Cochlear hypoplasia, enlarged vestibular aqueduct, and facial nerve anomaly were seen in all of the patients (Fig. 1). In all patients, cochlea demonstrated less than two turns consistent with cochlea hypoplasia type III (Fig. 1A) [20]. The modiolus was present but defective in all patients (Fig. 1D). The enlarged vestibular aqueduct was often observed as a circular shape with a diameter significantly larger than the posterior semicircular canal in the axial section of the temporal bone CT, which differed from the characteristic funnel-shaped enlargement of vestibular aqueduct seen in patients with *SLC26A4* mutations (Fig. 1B, Fig. S1). This finding could be related to the pathologic condition in which the main portion of enlargement is the endolymphatic duct rather than the endolymphatic sac. The MRI performed in 4 of the patients supported this speculation demonstrating bilateral dilation of the endolymphatic duct without enlarged endolymphatic sac in two patients and with unilateral mildly enlarged endolymphatic sac in two patients (Table 3, Fig. S1). The facial nerve ran inferior to the hypoplastic cochlea and displayed an obtuse angle between the labyrinthine and tympanic segments (Fig. 1C). Bulbous or funnel-shaped internal auditory canals were identified in 5 patients (50%). The vestibule was dilated bilaterally in all patients and severe lateral semicircular canal dysplasia was found unilaterally in one of the patients (patient 2). The ossicular chain was malformed or malpositioned to variable degrees in all of the patients, and the fusion of the malleoincudal joint and/or ossicular ankylosis to the epitympanic wall were present in five patients (patients 1–4, 9) (Fig. 1E). None of the patients exhibited obliteration of the oval window or round window on temporal bone CT.

**Table 3:** Radiologic findings in patients with BO/BOR syndrome.

Patients	TBCT						MRI	
	Cochlea	Vestibule	VA	ME	FN	IAC	ED	ES
1	CH (B)	Dilated (B)	Enlarged (B)	Ossicular anomaly (B)	Deviated (B)	Bulbous (B)		
2	CH (B)	Dilated (B), LSCC dysplasia (L)	Enlarged (B)	Ossicular anomaly (B)	Deviated (B)	–		
3	CH (B)	Dilated (B)	Enlarged (B)	Ossicular anomaly (B)	Deviated (B)	–		
4	CH (B)	Dilated (B)	Enlarged (B)	Ossicular anomaly (B)	Deviated (B)	–		
5*	CH (B)	Dilated (B)	Enlarged (B)	Ossicular anomaly (B)	Deviated (B)	Funnel (R)	Enlarged (B)	-
6*	CH (B)	Dilated (B)	Enlarged (B)	Ossicular anomaly (B)	Deviated (B)	–	Enlarged (B)	Enlarged (R)
7*	CH (B)	Dilated (B)	Enlarged (B)	Ossicular anomaly (B)	Deviated (B)	–	Enlarged (B)	Enlarged (L)
8	CH (B)	Dilated (B)	Enlarged (B)	Ossicular anomaly (B)	Deviated (B)	Bulbous (L)		
9	CH (B)	Dilated (B)	Enlarged (B)	Ossicular anomaly (B)	Deviated (B)	Bulbous (B)		
10*	CH (B)	Dilated (B)	Enlarged (B)	Ossicular anomaly (B)	Deviated (B)	Bulbous (L)	Enlarged (B)	-

* Patients who performed temporal MRI; TBCT = temporal bone computed tomography; MRI = temporal magnetic resonance imaging; CH = cochlear hypoplasia; VA = vestibular aqueduct; ME = middle ear; FN = facial nerve; IAC = internal auditory canal; ED = endolymphatic duct; ES = endolymphatic sac; LSCC = lateral semicircular canal.

### Auditory Manifestations

Serial pure tone audiometries revealed variable patterns of hearing loss. All patients presented with mixed type of hearing loss ranging from moderate to profound degree (Table 1). The onset was mostly perilingual or postlingual although congenital hearing loss was demonstrated in two of the patient. Five of 10 patients presented variable degrees of progressive hearing loss. Two of these patients showing progressive hearing loss experienced sudden aggravation of hearing loss, one of whom improved hearing after steroid treatment (patient 10) while the other became profoundly deaf without significant improvement despite steroid treatment (patient 7). Of the five patients showing stable hearing loss without significant progression over a period of two to four years, four patients were members of a single family.

### Middle Ear Surgeries and Auditory Rehabilitation

For auditory rehabilitation, five of 10 patients underwent middle ear surgeries (Table 1). In patient 5, ossiculoplasty was initially performed on the right ear to correct the conductive component of hearing loss, during which incudostapedial joint was found separated. Partial ossicular replacement prosthesis (PORP) was inserted between the stapes and malleus handle. However, revision ossiculoplasty was performed after 8 months due to failure of hearing gain and the location of the previously inserted PORP was adjusted. Persistence of the air-bone gap led to middle ear exploration, and stapedotomy was performed because of impaired stapes mobility. Despite multiple middle ear surgeries, hearing gain could not be achieved and the patient is using hearing aids for auditory rehabilitation. Bone conduction hearing was slightly worsened on both sides after multiple surgeries, which was not thought to be the result of middle ear manipulations. In patient 6, incudostapedial joint was found fixed during middle ear exploration, which led to the insertion of PORP after the removal of incus. One year later, revision ossiculplasty was performed to reposition the PORP but failed to close the air-bone gap. Subsequently, cochlear implantation was performed on the same side, which improved her hearing and language ability. During cochlear implantation, the facial nerve dehiscence was seen at the tympanic segment which ran anterosuperiorly to the stapes passing superior to the round window. Perilymphatic gusher occurred after cochleostomy but was easily controlled by conventional methods. Two other patients (patients 8 and 10) had a history of receiving two ossiculoplasties of the same ear and stapedotomy of both ears, respectively, at other hospitals. Mixed hearing loss was still observed in both of these patients despite middle ear operations at the time of initial visit to our clinic. In another patient (patient 9), a congenital cholesteatoma filling middle ear space eroding incus and malleus was identified, and incus interposition was performed after removal of cholesteatoma. Closure of air-bone gap also failed in this patient who is currently using hearing aids on both sides for auditory rehabilitation.

### Mutation Analysis

**Figure 2:**
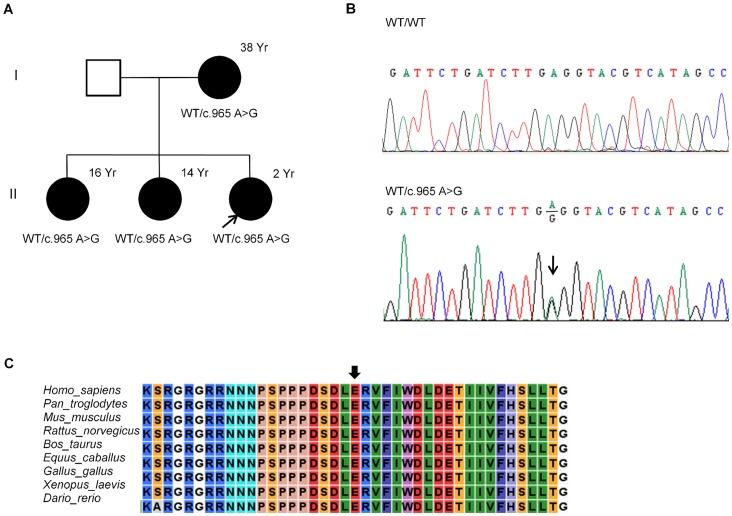
Identification of a novel missense mutation in the *EYA1* gene. (A) Pedigree of family I comprised of two generations. Squares and circles indicate females and males, respectively. Filled symbols display affected individuals and the arrow appoints the proband of the family. The ages of the affected females are marked in years (Yr) next to the symbols. (B) Nucleotide sequence of exon 10 of the *EYA1* gene. Black arrow indicates the heterozygous nucleotide substitution, c.965 A>G, detected in the affected family members. (C) Multiple alignments of the *EYA1* homologous sequences of nine different vertebrates. The amino acid substituted by the missense mutation p.E332G (arrow) is highly conserved among the different vertebrate species.

**Figure 3:**
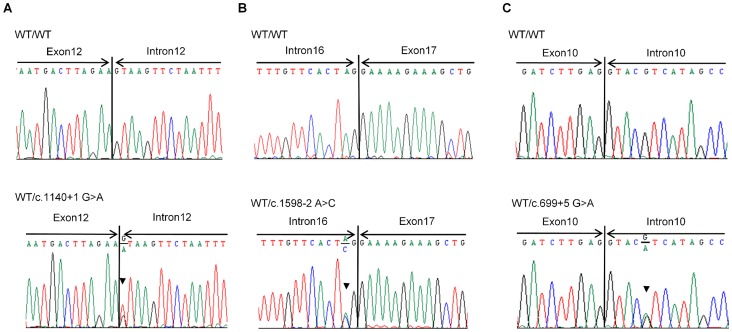
Identification of three novel splice site mutations in the *EYA1* gene. The nucleotide sequences of the control (WT/WT) and the affected individuals (WT/MT) are shown for patients 5 (A), 6 (B), and 7 (C). Each heterozygous mutation is indicated by arrowheads. (A, C) The mutations c.1140+ G>A and c.699+5 G>A are splicing donor site mutations of exons 12 and 10, respectively. (B) The mutation c.1598-2G>A is splicing acceptor site mutation of exon 17.

All exons and exon-intron boundaries of *EYA1*, *SIX1*, and *SIX5* genes were sequenced in 7 families with BOR/BO syndrome. One missense and three splice site mutations were identified in *EYA1*, while no mutations were found in either *SIX1* or *SIX5* gene (Table 4–5). A novel missense mutation, p.E332G, was identified in all four affected members of Family I, which caused an adenine to guanine substitution at nucleotide position 965 converting glutamic acid to glycine (Fig. 2A–B). This mutation changed the charge of amino acids from a negative charge to nonpolar. The conservation of the mutated amino acid was analyzed using the CLC sequence viewer, which demonstrated that the glutamic acid at amino acid position 332 is highly conserved in various vertebrates (Fig. 2C). Three novel *EYA1* splice site mutations, c.1140+1 G>A, c.1598-2 A>C and c.699+5 G>A, were detected in 3 other families (Table 4, Fig. 3).

**Table 4:** Five novel mutations identified in *EYA*1 gene.

Families	Patients	Location	Nucleotide change	Amino acid change	Type
I	1–4	Exon 10	c.965 A>G	p.E322G	Missense
II	5	Intron 12	c.1140+1 G>A	–	Splice site
III	6	Intron 16	c.1598-2 A>C	–	Splice site
IV	7	Intron 10	c.699+5 G>A	–	Splice site
V	8	Whole gene	Large deletion		

**Table 5:** Non-pathogenic polymorphisms detected in *EYA1* and *SIX5* genes.

Gene	Location	Nucleotidechange	Amino acidchange	Reference
*EYA1*	Intron 3	c.125-169T>C	–	rs.7840811
	Intron 7	c.556+78T>A	–	–
	Intron 8	c.639+39T>G		rs3779747
	Exon 9	c.813A>G	p.T271T	rs1445398
	Intron 11	C.1050+107A>G	–	rs76660214
	Intron 11	c.1050+113G>A	–	rs2053664
	Exon 14	c.1278C>T	p.G426G	rs4738118
	Intron 14	c.1360+53C>T	–	rs4737312
	Intron 15	c.1476-21G>T	–	rs3735935
	Intron 17	c.1699-55G>A	–	rs10103644
	Intron 17	c.1699-23A>G	–	rs10090382
	Exon 18	c.1755T>C	p.H585H	rs10103397
	Intron 18	c.*509_*512delAAAA	–	rs146202037
	Intron 18	c.*1324T>C	–	rs56115941
	Intron 18	c.*1581G>A	–	rs9298163
*SIX5*	Intron 1	C.803+123C>A	–	rs3745802
	Exon 3	c.1903C>T	p.P635S	rs.2014576
	3′ UTR	c.*34G>A	–	–

### Splicing Assay

**Figure 4:**
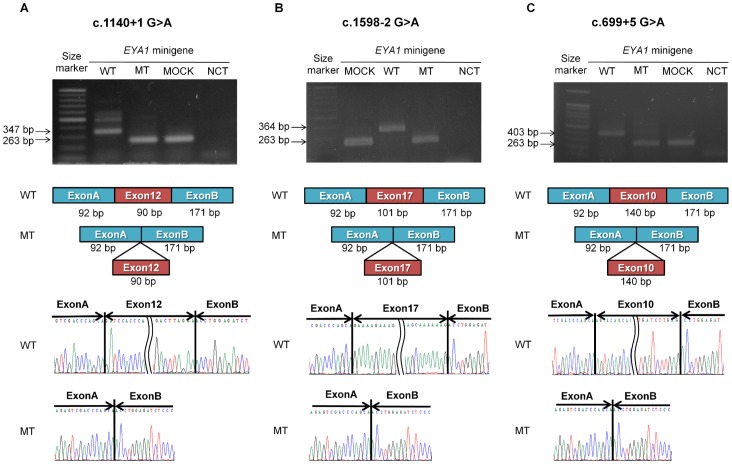
Splicing assays for the three novel splice site mutations in the *EYA1* gene. The size of the amplified product of *EYA1* minigene transcripts of splice site mutations c.1140+1 G>A (A), c.1598-2 G>A (B), and c.699+5 G>A (C), were 263 bp which is smaller than that of the wild type, suggesting exon skipping. The different splicing processes of the wild type and mutant transcripts are shown in a schematic illustration. Partial nucleotide sequences of the wild and mutant types are presented. MOCK, MOCK vector; WT, wild type; MT, mutant type; NCT, negative control.

To investigate the potential pathogenic effect of the three novel splice site mutations (c.1140+1 G>A, c.1598-2 A>C and c.699+5 G>A) on normal splicing, each exon and the flanking intronic sequences sufficient to allow splicing were inserted into a pSPL3 or pSPL3b vector. Each vector was transfected into the HeLa cells, and transcribed to mRNA in the cells. All three splice site mutations were found to disrupt the normal splicing (Fig. 4). Exons 12 (90 bp), 17 (101 bp), and 10 (140 bp), involved with mutations c.1140+1 G>A, c.1598-2 A>C, and c.699+5 G>A, respectively, were inserted between exon A (92 bp) and exon B (171 bp) of the pSPL3 or pSPL3b vector. The size of the mutant mRNA was 263 bp which included only exons A and B of the vector, meaning that exon skipping occurred for all three splice site mutations (Fig. 4). These results were confirmed by Sanger sequencing showing that 263 bp of the mutant mRNA contained only exons A and B of the vector (Fig. 4).

### Identification of *EYA1* Deletion

**Figure 5:**
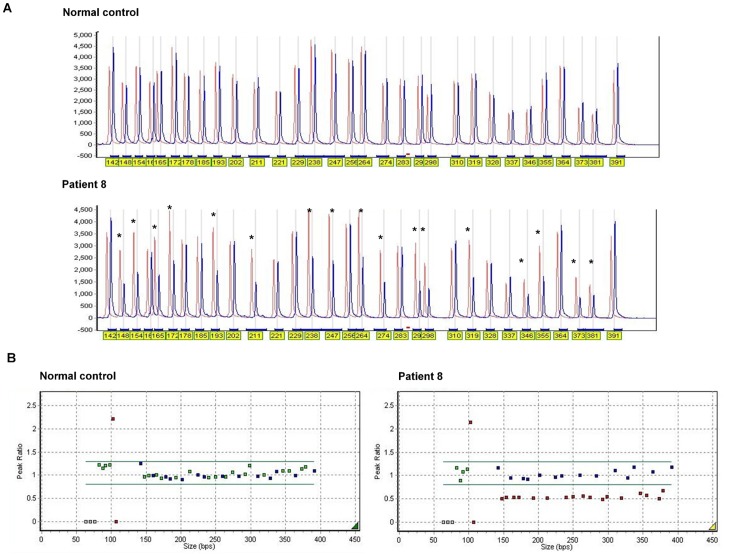
Multiplex Ligation-dependent probe Amplification (MLPA) analysis for the detection of deletions in the *EYA1* gene. (A) Comparison of the relative peak height between the reference probes (red peak) and the EYA1 probes (blue peak) in normal control and patient 5. The asterisks indicate the control probes located in other chromosomal regions. (B) The normalization ratio in a normal control and patient 5 to reference samples. The two green horizontal lines indicate the cut-off normalization values for loss (0.8) or gain (1.2) of relative copy numbers. The blue dots mark the values of the reference probes whereas green and red dots indicate *EYA1* probes either within or beyond the normal range, respectively.

The MLPA analysis was performed in three patients (patients 8–10) who were found not to carry any mutations in the three genes (*EYA1*, *SIX1* and *SIX5*) by direct sequencing, and the results were compared to the data acquired in one normal control with normal hearing confirmed by pure tone audiometry. The entire *EYA1* gene was suspected to be deleted in patient 8 (Fig. 5). In this patient, the peak height of all *EYA1* probes was lower than half of the reference peak, and the normalization ratio was approximately 0.5 compared with the reference values. In contrast, all 14 control probes of the same patient located in other chromosomal regions demonstrated values same as the reference peak with suitable normalization ratios (0.8∼1.2). The results of the MLPA indicated that patient 8 had a heterozygous deletion encompassing the whole *EYA1* gene. To investigate the deleted region including *EYA1* in patient 8, genotype analysis using microsatellite markers was performed. Only patient 8 revealed homozygous alleles for all 4 markers, while all 12 normal controls showed heterozygous alleles for at least one of the microsatellite markers (Table S2). In addition, genotypes for all 15 polymorphisms detected in the *EYA1* gene were homozygous in patient 8 (Table 5). These results strongly suggested that patient 8 carried a large deletion including the whole *EYA1* gene, although the range of the deleted chromosomal region could not precisely be estimated.

## Discussion

In this study, 5 of 7 unrelated Korean families clinically diagnosed as BOR/BO syndrome were identified to carry mutations in the *EYA1* gene, including four novel intragenic mutations and one large deletion encompassing the whole *EYA1* gene. Pathogenic variations of the *SIX1* and *SIX5* genes were not found in any of the patients. Clinically, auditory manifestations known as the most common and characteristic feature of BOR/BO syndrome were analyzed in detail together with the outcome of various treatment modalities for hearing improvement such as middle ear surgeries, cochlear implantations, or hearing aids.

In contrast to the western population where large cohort studies have been performed on BOR/BO syndrome, limited information is available concerning genetic mutations of this syndrome in the East Asian population, and a total of 16 mutations in the *EYA1* gene have been reported including 7 nonsense, 3 frameshift, 3 splice-site, 2 missense mutations, and 1 partial deletion [17]. In this study, *EYA1* mutations were identified in 71% (5 of 7 families) which is higher than 40% previously reported by Chang *et al.* [3]. Splice site mutations were the most common type found in 3 of 7 families, followed by one missense mutation and one large deletion encompassing the whole *EYA1* gene. In the Korean population, there have been only 3 case reports of patients with BOR/BO syndrome carrying *EYA1* mutations, and this study adds to the genotypic and phenotypic spectrum of BOR syndrome in the East Asian population [15], [21], [22]. As for *SIX1* mutations associated with BOR/BO syndrome, more than 10 mutations have been identified worldwide, while there is only one missense mutation reported in the East Asian population [5], [7], [9], [12], [23], [24]. Consistently, no *SIX1* or *SIX5* mutation was found in this study suggesting the limited role of *SIX* genes as the cause of BOR/BO syndrome in the East Asian population. Since a large deletion was identified by MLPA in one patient with BOR/BO syndrome, we suggest that additional studies should be performed to identify complex rearrangements or large deletions when no mutation is found by conventional sequencing techniques.

No clear genotype-phenotype correlation could be demonstrated in this study in accordance with previous reports, in which no mutation was found in a patient with all major features of BOR syndrome (patient 9) whereas a splice site mutation of the *EYA1* gene was identified in a patient presenting with only mixed hearing loss and enlarged vestibular aqueduct (patient 7) [7]. Also, no definite difference could be identified regarding the severity of clinical features of BOR/BO syndrome or the presence of additional phenotypes in the patient who carried a large deletion encompassing the *EYA1* gene (patient 8) compared to the patients with intragenic *EYA1* mutations.

The progression of hearing loss was variable in patients with BOR/BO syndrome included in this study. Cremers *et al.* [19] have reported that the hearing loss in BOR/BO syndrome was stable without progression or fluctuation. However, half of the patients included in this study demonstrated various degrees of progressive hearing loss, and also sudden aggravation of hearing was demonstrated in one patient. Kemperman *et al.* [25] have also reported a patient with BOR syndrome showing progressive hearing loss and suggested the correlation between the presence of enlarged vestibular aqueduct and progressive fluctuant hearing loss. In developmental studies, the expression of murine *eya1* in the sensory hair cells continued after birth and after maturation (P16 in mouse), suggesting an additional role for eya1 in the differentiation and/or survival of the inner ear cell populations in particular the sensory cells [26]. This may provide evidence for the progression of hearing loss demonstrated in some of the patients with *EYA1* mutations considering the possible role of EYA1 in the maintenance and survival of the hair cells and the supporting cells. Therefore, we believe that the possibility of hearing progression should be explained to the patients with BOR/BO syndrome, and regular auditory tests should be performed in order to treat these patients with proper modality of auditory rehabilitation at an appropriate timing.

This study clearly demonstrated the limitation of middle ear surgeries for hearing improvement in patients with BOR/BO syndrome consistent with previous reports [19]. The failure may be explained by several reasons. First, complex multiple anomalies of the middle ear may have caused the persistence of air-bone gap after simply reconstructing or modifying the ossicular chain by performing ossiculoplasty or stapedotomy. Embryologically, murine *eya1* has been reported to be expressed in the mesenchyme surrounding the cartilage premordia of all three ossicles and also in the epithelium of the tubotympanic recess which later develops into the tympanic cavity at E13.5, meaning that mutation in the *EYA1* gene can disrupt normal development of the middle ear in various aspects [26]. Decreased middle ear space, anomalies of the oval and round windows, together with the abnormal angle of the ossicles can all act as hindering factors for successful middle ear surgeries. For example, abnormal angle of the stapes relative to the tympanic membrane or abnormal position of the incus relative to the stapes footplate can interrupt the proper positioning of the middle ear prosthesis resulting in disturbance of sound transmission to the inner ear. Even in limited cases showing successful hearing gain after middle ear surgeries, recurrence of air-bone gap has been reported to occur, which may be related to the unstable placement of the middle ear prosthesis inevitably caused by the multiple structural abnormalities of the ossicles and the middle ear cavity [19]. Secondly, vestibular aqueduct enlargement that was seen in all of the patients in this study may have acted as a third window causing the air-bone gap. As reported in previous studies, air-bone gap caused by the third window effect cannot be improved by middle ear explorations [27], [28]. Third reason could be related to the defective modiolus and enlarged internal auditory canals often seen in these patients, which can be speculated to cause increased perilymphatic pressure resulting in decreased mobility of stapes and reduction of sound transmission. Since typical clinical features of BOR/BO syndrome other than hearing loss can be easily overlooked before performing initial middle ear explorations and unsuccessful outcome may be encountered unexpectedly, careful review of CT findings and history taking as well as thorough physical examinations should always be performed in patients showing mixed hearing loss. In the future, patients with BOR/BO syndrome may be good candidates of active middle ear implants considering the failure of hearing gain by conventional middle ear reconstruction techniques.

Since patients failed to gain hearing after one or more middle ear surgeries, most of the patients in this study used bilateral hearing aids. In two of the patients with severe to profound hearing loss who could not benefit from hearing aids, cochlear implantation was performed. In patients with syndromic hearing loss, multiple factors have to be considered regarding auditory rehabilitation, including combined mental retardation and developmental delay in addition to various inner ear malformations. Cochlear implantation in patients with syndromic hearing loss is challenging in both surgical and audiologic aspects. Although good results of cochlear implantation have been reported in these patients, some of the syndromes associated with cochlear nerve deficiency or narrow internal auditory canals such as CHARGE syndrome have shown limited outcome [29], [30]. Although the *EYA1* gene is involved in the development of the spiral ganglion and cochlear hypoplasia often seen in patients with BOR/BO syndrome has been known to be a poor prognostic factor of cochlear implantation, successful outcome in terms of speech and auditory performances could be achieved in our patients after cochlear implantation [26], [31]. In addition, no surgical complications were encountered despite multiple inner ear and facial nerve anomalies. Since there is limited data on the outcome of cochlear implantation in patients with syndromic hearing loss, especially BOR/BO syndrome, the results of this study may provide some evidence for recommending cochlear implantation in patients with BOR/BO syndrome who cannot benefit from hearing aids despite the conductive component of hearing loss.

### Conclusions

Considering the high mutation rate of the *EYA1* gene in Korean patients with BOR/BO syndrome, the mutational analysis of *EYA1* should be an integral part of the diagnosis of BOR/BO syndrome in the East Asian population. The characteristic inner ear and middle ear anomalies and mixed type hearing loss may also provide clinical clues to suspect BOR/BO syndrome even in the absence of other typical clinical features. The management of hearing loss and auditory rehabilitation in BOR/BO syndrome should be individually tailored keeping in mind the high failure rate of hearing gain achieved by middle ear explorations in patients with mixed hearing loss. Hearing aids are good options in patients with mild to severe hearing loss, but regular hearing evaluations are needed considering the possibility of progression of hearing loss in order to treat these patients with proper modality of auditory rehabilitation at an appropriate timing. Successful outcome can be expected with cochlear implantations in patients with BOR/BO syndrome who cannot benefit from hearing aids. The novel *EYA1* mutations identified in this study adds to the genotypic and phenotypic spectrum of BOR syndrome in the East Asian population and the clinical results of this study may provide evidence for recommending proper means of auditory rehabilitation in patients with BOR/BO syndrome.

## Supporting Information

Figure S1 **Temporal bone CT and temporal MRI findings of patient 10 demonstrating enlarged vestibular aqueduct in a circular shape.** (A–F) Axial view of temporal bone CT shows enlarged vestibular aqueduct (black arrows) observed as a circular shape with a diameter significantly larger than that of the posterior semicircular canal (black arrowhead in Fig. S1A). (F–K) Axial view of temporal MRI also exhibited enlargement of the endolymphatic duct (white arrows) whereas the endolymphatic sac was not enlarged (white arrowhead in Fig. S1K).
(TIF)

Table S1 **Primer information of EYA1, SIX1 and SIX5.**
(DOC)

Table S2 **Identification of** ***EYA1*** **deletion by microsatellite marker.**
(DOC)
