# Supplementary figures and images for: Mutational Analysis of EYA1, SIX1 and SIX5 Genes and Strategies for Management of Hearing Loss in Patients with BOR/BO Syndrome
Source: PLoS One. 2013 Jun 28;8(6):e67236. doi: 10.1371/journal.pone.0067236 (PMC3696009; doi:10.1371/journal.pone.0067236)

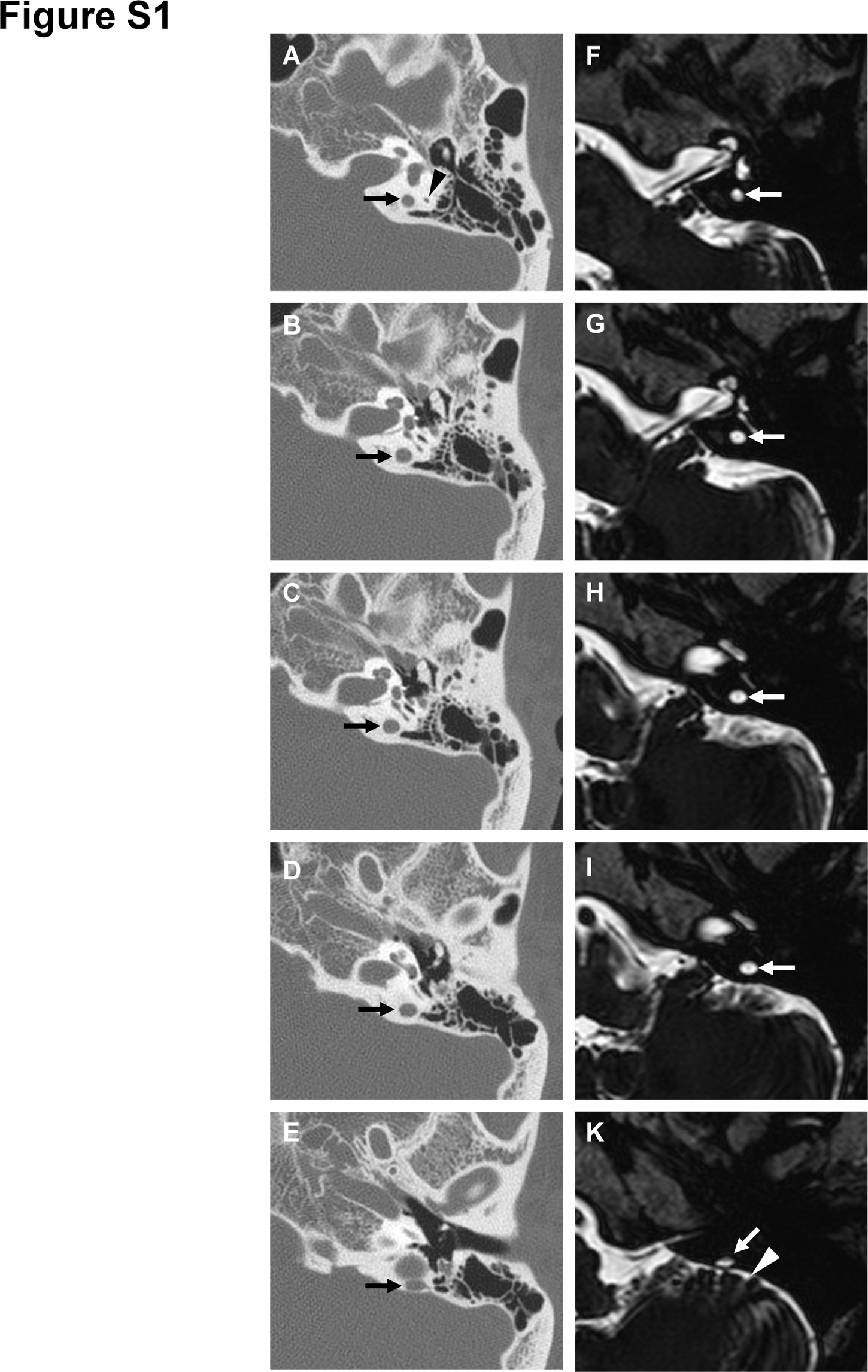

Supplement: Figure S1 — Temporal bone CT and temporal MRI findings of patient 10 demonstrating enlarged vestibular aqueduct in a circular shape. (A–F) Axial view of temporal bone CT shows enlarged vestibular aqueduct (black arrows) observed as a circular shape with a diameter significantly larger than that of the posterior semicircular canal (black arrowhead in Fig. S1A). (F–K) Axial view of temporal MRI also exhibited enlargement of the endolymphatic duct (white arrows) whereas the endolymphatic sac was not enlarged (white arrowhead in Fig. S1K). (TIF) [file pone.0067236.s001.tif]
