# Supplementary material for: Mutational Analysis of EYA1, SIX1 and SIX5 Genes and Strategies for Management of Hearing Loss in Patients with BOR/BO Syndrome
Source: PLoS One. 2013 Jun 28;8(6):e67236. doi: 10.1371/journal.pone.0067236 (PMC3696009; doi:10.1371/journal.pone.0067236)
Supplement: Table S1 — Primer information of EYA1, SIX1 and SIX5. (DOC) [file pone.0067236.s002.doc]

Table S1. Primer information of EYA1, SIX1 and SIX5.

| Gene | Primer | Forward | Reverse | Product size(bp) | Annealing  Temperature |
| --- | --- | --- | --- | --- | --- |
| *EYA1* | Exon1 | 5’-AGAGGACGCGTGTGTGTTGA-3’ | 5’-AAAGCGCTCCTGTTAGCTTGG-3’ | 827 | 62 |
|  | Exon2 | 5’-CACGATCATTTTGTCATTCG-3’ | 5’-TTTCCAACAGAGGCTGTTACT-3’ | 538 | 57 |
|  | Exon3 | 5’-TGTGCAAGTGTGTTTCAAAGG-3’ | 5’-GCATTTGGTGAAACGAAACC-3’ | 446 | 58 |
|  | Exon4 | 5’-TTGTTTCCTAAGGGGAAAGAAC-3’ | 5’-TGTGACTCTGAGCAAAGCTATTTT-3’ | 449 | 53 |
|  | Exon5 | 5’-TTGCGAAGATTGTGATGACG-3’ | 5’-GGAACATGTGGGCACAGAC-3’ | 403 | 58 |
|  | Exon6 | 5’-GCTGGAATTTGTGATGTGGTT-3’ | 5’-ATTGGCCAAAGATTGGGTCT-3’ | 329 | 58 |
|  | Exon7 | 5-‘TGAGATAAGATTGGGGAAGCA-3’ | 5’-CAGGTGTCCTGCCTCTAAGC-3’ | 443 | 58 |
|  | Exon8 | 5’-CCAAATACCAATTCTGCCTTTT-3’ | 5’-TGAAAACCAAACAACTCACCA-3’ | 345 | 58 |
|  | Exon9 | 5’-GTACTTGTATGCCCCCGTGT-3’ | 5’-CCAAAATTGTGCAACCACTG-3’ | 417 | 58 |
|  | Exon10 | 5’-ACCAGCGCAAGTAAAAGACG-3’ | 5’-GCATCTGATACCTTAACCACTGC-3’ | 390 | 58 |
|  | Exon11 | 5’-TCATTCATCTTCCGTTTCAAGA-3’ | 5’-CACTGGGGTCTGAATAAGCA-3’ | 434 | 58 |
|  | Exon12 | 5’-CATCAACATTTGGGGCTCTT-3’ | 5’-AGGCAAAACACATTGCCATA-3’ | 444 | 58 |
|  | Exon13 | 5’-GACTGCCACCTACTGATTGACA-3’ | 5’-GGAAAGCCATCTGTTCCAAA-3’ | 341 | 58 |
|  | Exon14 | 5’-AAGGTGAGCACCCTTGAATG-3’ | 5’-GGCCAGTGAGATGAAACTG-3’ | 406 | 58 |
|  | Exon15-16 | 5’-CAAAGCCGAAGAAATATGTTG-3’ | 5’-TCCTGAAGGAAAAGAGCTGA-3’ | 450 | 58 |
|  | Exon17 | 5’-GCATTCGAATCAGAGCATGA-3’ | 5’-CTTGTGGCCCTTGAGTTTG-3’ | 474 | 58 |
|  | Exon18-1 | 5’-GTCAGCATGGGAGTGGATTT-3’ | 5’-AGACGTGGTCCTCCATTCAC-3’ | 367 | 58 |
|  | Exon18-2 | 5’-TCTGTGACCAGGGACAGAT-3’ | 5’-TGTACTTGCCATGTTGTGAG-3’ | 597 | 57 |
|  | Exon18-3 | 5’-ACTTTTTGGTGTCTCATACCC-3’ | 5’-GGTGGTAGGAGAGCTCATTT-3’ | 625 | 57 |
|  | Exon18-4 | 5’-AAGCATGGATGTCAAGTGTC-3’ | 5’-cAAACTGACCACTTTTGGAG-3’ | 563 | 57 |
|  | Exon18-5 | 5’-ATTGTCCATCACCATGGAAAC-3’ | 5’-TGTTACCCCTGCAAGTTCAGT-3’ | 713 | 55 |
|  |  |  |  |  |  |
| *SIX1* | Exon1 | 5’-AAGTTCCGACTCCGGTTTTC-3’ | 5’-CCATCCTTAGCAAGGAACCTC-3’ | 900 | 58 |
|  | Exon2 | 5’-AGGGTAGAGCTCCTCCATTTG-3’ | 5’-AAGACGCAAAAAGTTCCTGA-3’ | 547 | 55 |
|  | Exon1-1 | 5’-GGGACGCAAGCTCGAGAAAA-3’ | 5’-AGGCAAGGTAGCCATGTTT-3’ | 564 | 62 |
|  |  |  |  |  |  |
| *SIX5* | Exon1-2 | 5’-TACAAAACATGGCTACCTTGC-3’ | 5’-GGAACTTCTTGCGCAGTCGAT-3’ | 585 | 60 |
|  | Exon1-3 | 5’-AAGTGTCGACTGCGCAAGAAG-3’ | 5’-CTGTCCCGTCCACACTTAGTC-3’ | 425 | 60 |
|  | Exon2-1 | 5’-TGGAGTTTGGAGTGGTCTCA-3’ | 5’-CCAGAGAGGTCTTGGTCTCG-3’ | 500 | 62 |
|  | Exon2-2 | 5’-CAGCGAGACCAAGACCTCTC-3’ | 5’-CCAAGCCAGAGAGAAGTGGA-3’ | 670 | 62 |
|  | Exon3-1 | 5’-TCCACTTCTCTCTGGCTTGG-3’ | 5’-CTGTTCCTGCGCTTAGTTCC-3’ | 484 | 60 |
|  | Exon3-2 | 5’-AGAGGGGCTGATGTTGTCAC-3’ | 5’-GGGTGTTCCGCTTACAGCTA-3’ | 585 | 55 |
|  | Exon3-3 | 5’-CCATATACGTGGGGGACTCA-3’ | 5’-CTGAATGCAAACCCCTCTTC-3’ | 591 | 55 |
