# Supplementary material for: Mutational Analysis of EYA1, SIX1 and SIX5 Genes and Strategies for Management of Hearing Loss in Patients with BOR/BO Syndrome
Source: PLoS One. 2013 Jun 28;8(6):e67236. doi: 10.1371/journal.pone.0067236 (PMC3696009; doi:10.1371/journal.pone.0067236)
Supplement: Table S2 — Identification of EYA1 deletion by microsatellite marker. (DOC) [file pone.0067236.s003.doc]

Table S2. Identification of *EYA1* deletion by microsatellite marker.

| Sample | D8S1795 | D8S1060 | D8S1807 | D8S570 |
| --- | --- | --- | --- | --- |
| **Patient 8** | **1/1** | **1/1** | **4/4** | **2/2** |
| Patient 9 | 1/2 | 1/1 | 1/6 | 6/6 |
| Patient 10 | 2/2 | 1/1 | 4/4 | 6/6 |
| Normal control 1 | 4/4 | 1/1 | 2/4 | 4/5 |
| Normal control 2 | 2/3 | 1/1 | 2/3 | 5/6 |
| Normal control 3 | 3/6 | 1/1 | 3/6 | 6/8 |
| Normal control 4 | 2/2 | 1/1 | 4/6 | 1/7 |
| Normal control 5 | 2/2 | 1/1 | 6/6 | 6/7 |
| Normal control 6 | 2/6 | 1/1 | 2/5 | 6/6 |
| Normal control 7 | 2/2 | 1/1 | 3/3 | 2/7 |
| Normal control 8 | 2/5 | 1/1 | 5/6 | 6/8 |
| Normal control 9 | 2/4 | 1/1 | 2/4 | 6/6 |
| Normal control 10 | 2/5 | 1/1 | 6/7 | 6/6 |
| Normal control 11 | 2/2 | 1/1 | 3/4 | 6/6 |
| Normal control 12 | 2/2 | 1/1 | 3/3 | 6/7 |
